# Pesticide exposure and cardiovascular health in non-CVD mortality population: novel evidence from NHANES 2007–2018 using Life's Essential 8

**DOI:** 10.3389/fnut.2025.1578796

**Published:** 2025-06-26

**Authors:** Jiang He, Hexilin Wang, Xia Li

**Affiliations:** ^1^Department of Hypertension and Vascular Disease, The First Affiliated Hospital of Sun Yat-sen University, National-Guangdong Joint Engineering Laboratory for Diagnosis and Treatment of Vascular Diseases, Key Laboratory on Assisted Circulation, Ministry of Health, Guangzhou, China; ^2^Department of Cardiology, The First Shenyang Hospital of China Medical University, Shenyang, Liaoning, China

**Keywords:** pesticide exposure, Life's Essential 8, cardiovascular health, NHANES, cardiometabolic index, inflammatory markers, mixed exposure, dose-response relationship

## Abstract

**Background:**

While pesticide exposure has become a global public health concern, its impact on cardiovascular health among non-cardiovascular mortality populations remains understudied.

**Methods:**

Based on NHANES 2007–2018 data (*n* = 12,432), we examined associations between five pesticide biomarkers (2,4-D herbicides, atrazine metabolites, organophosphate dimethyl and diethyl metabolites, glyphosate) and cardiovascular health, assessed by Life's Essential 8 (LE8) scores and Cardiometabolic Index (CMI). The associations were explored through multiple regression analyses, weighted quantile sum (WQS) regression, restricted cubic spline (RCS) analysis, and mediation analysis.

**Results:**

2,4-D herbicides showed significant negative correlations with multiple LE8 components, particularly in BMI scores (β = −1.441, 95% CI: −2.158, −0.725) and diet scores (β = −1.241, 95% CI: −1.825, −0.658). Organophosphate metabolites demonstrated positive associations with smoking and diet scores. Dose-response analysis revealed an inverted U-shaped relationship between 2,4-D and LE8 scores, while organophosphates showed consistent positive correlations. WQS regression indicated that glyphosate contributed most significantly to LE8 scores (58%), while organophosphate diethyl metabolites dominated CMI effects (62%). Inflammatory markers (CRP and SII) played crucial roles in mediating pesticide exposure's effects on cardiovascular health.

**Conclusion:**

This study provides the first systematic evidence of association patterns between pesticide exposure and cardiovascular health in the general population, revealing differential impacts across pesticide types. These findings provide important scientific basis for understanding pesticide exposure's health effects and developing targeted prevention strategies.

## 1 Introduction

Pesticide exposure has emerged as a global public health concern. In developing countries, health issues related to pesticides are particularly severe due to inadequate protective measures and regulatory systems, with estimated annual pesticide-related deaths exceeding 160,000. Epidemiological studies have linked pesticide exposure to various chronic conditions, including neurological disorders, endocrine disruption, and cardiovascular diseases (1, 2).

Numerous epidemiological studies have examined the relationship between pesticide exposure and cardiovascular mortality. A systematic review encompassing 24 articles demonstrated an association between pesticide exposure and cardiovascular disease (3). Additionally, a cohort analysis of 7,557 Japanese-American males revealed that high-level occupational pesticide exposure correlated with cardiovascular disease incidence over a 10-year period (Hazard Ratio = 1.46, 95% CI = 1.10–1.95, *P* = 0.009) (4). Animal studies have provided crucial evidence for understanding the underlying biological mechanisms. In zebrafish models, permethrin was found to cause cardiac enlargement, accelerated heart rate, significantly increased blood flow velocity, and elevated metabolic rates; furthermore, it was discovered to interact with several ion channels, inducing changes in cardiovascular-related markers (5). Rodent studies further confirmed that pesticide exposure can increase oxidative stress and oxidative modifications of genomic DNA content in rabbit cardiac tissue (6). These findings provide mechanistic explanations for the association between pesticide exposure and cardiovascular disease. However, the potential associations between pesticide exposure and non-cardiovascular mortality, and their impact on cardiovascular health in this population, remain systematically understudied, representing a critical knowledge gap.

Life's Essential 8 (LE8) and the Cardiometabolic Index (CMI) are vital tools for assessing cardiovascular health. Introduced by the American Heart Association in 2022, LE8 is a novel assessment system encompassing eight dimensions: diet, physical activity, nicotine exposure, sleep health, body weight, blood lipids, blood glucose, and blood pressure (7, 8). CMI provides a comprehensive perspective on metabolic health status by combining the triglyceride to high-density lipoprotein cholesterol ratio (TG/HDL-C) with the waist-to-height ratio (WHtR). A longitudinal cohort study confirmed CMI's significant association with cardiovascular disease mortality risk (9). Both indicators have demonstrated significant value in predicting cardiovascular events and assessing overall health outcomes.

This study utilizes the National Health and Nutrition Examination Survey (NHANES) database to explore, for the first time, the relationship between pesticide exposure and non-cardiovascular mortality. Through analysis of multiple pesticide biomarkers (2,4-D, Atrazine, OP-Dimethyl, OP-Diethyl, and Glyphosate), we comprehensively evaluate the associations between pesticide exposure and non-cardiovascular mortality risk in the general population. NHANES's detailed pesticide metabolite data and long-term cause-of-death follow-up information (average follow-up exceeding 10 years) provide unique advantages for revealing the relationship between pesticide exposure and health outcomes. The findings of this study will not only offer new perspectives on understanding the health impacts of pesticide exposure but also provide crucial scientific evidence for developing targeted public health intervention strategies.

## 2 Materials and methods

### 2.1 Data source

This study conducted a cross-sectional analysis using the NHANES database (https://www.cdc.gov/nchs/nhanes/index.html), which contains comprehensive information about the health and nutritional status of U. S. citizens, along with data from studies tracking diseases and risk factors (10). The database encompasses basic information, dietary and nutritional details, physical examination results, laboratory findings, survey data, and other confidential information. The study examined data from six NHANES cycles: 2007–2008, 2009–2010, 2011–2012, 2013–2014, 2015–2016, and 2017–2018, initially comprising 59,842 individuals. During the data screening phase, individuals under 20 years of age (*n* = 25,072), cases with missing LE8-related information (*n* = 962), individuals lacking pesticide exposure data (n = 16,866), and cases with incomplete data (*n* = 4,510) were excluded. Following these exclusion criteria, the final study population consisted of 12,432 adult participants (Supplementary Figure S1).

### 2.2 Pesticide assessment

This study analyzed pesticide biomarker data from the NHANES database. Initially, 95 pesticide biomarkers across eight major categories were included (11):

1. Atrazine class: Atrazine and its 6 metabolites (e.g., Atrazine mercapturate, Desethyl atrazine),
2. DEET class: DEET and its 3 metabolites,
3. Organochlorine class: 14 compounds (including β-hexachlorocyclohexane, aldrin, dieldrin, chlordane, DDT, and their metabolites),
4. Organophosphate class: 6 phosphate ester metabolites (dimethyl and diethyl series) and 4 parent compounds,
5. Herbicides: 2,4-D and its 5 metabolites,
6. Neonicotinoid class: 6 compounds (including imidacloprid, thiamethoxam, and their metabolites),
7. Sulfonylurea class: 16 compounds (including metsulfuron, bensulfuron, etc.),
8. Other pesticide metabolites: 34 compounds (including phenoxybenzoic acids, trichloropyridinol, etc.).

The detectability percentage for target chemicals was calculated by dividing the total number of measurements above LOD (Limit of Detection) by the total number of measurements for that chemical in NHANES. To ensure inclusion of chemicals above detection limits for most study participants, we maintained a detection frequency percentage of 50% or higher across the entire population. Ten pesticide biomarkers were ultimately included in the analysis: herbicide 2,4-D and its metabolites (URX14D, URXDCB), atrazine metabolite (URXDEA), organophosphate pesticide metabolites (including dimethyl series URXOP1-3 and diethyl series URXOP4-6), and glyphosate metabolite (URXDHD). All biomarker concentrations were corrected for urinary creatinine.

The selection of these five specific pesticide biomarkers was based on several important criteria. First, these pesticides had consistently measurable levels across multiple NHANES cycles (2007–2018), with detection rates exceeding our 50% threshold, ensuring sufficient statistical power for analysis. Second, these pesticides represent the most commonly used agricultural and residential pesticides in the United States, with widespread human exposure documented in multiple NHANES cycles. Third, these compounds have distinct chemical structures and mechanisms of action, allowing us to evaluate potentially different pathways of cardiovascular impact. Fourth, extensive toxicological literature exists for these compounds, providing biological plausibility for potential cardiovascular effects. Finally, these selected pesticides represent different use patterns and exposure routes, providing a comprehensive assessment of the overall pesticide burden in the general population.

### 2.3 Covariates

The following variables were included in the analysis as confounding factors: age (20–39, 40–59, ≥60 years), sex (male and female), race/ethnicity (Mexican American, Other Hispanic, Non-Hispanic White, Non-Hispanic Black, Other), education level (less than high school, high school or equivalent, above high school), and family income-to-poverty ratio (< 1, 1–3, >3). Life's Essential 8 scores were categorized into low (< 50 points), moderate (50–79 points), and high (≥80 points) levels. Self-reported diseases primarily included cardiovascular diseases (heart failure, coronary heart disease, angina, heart attack, or stroke). All variables were derived from NHANES questionnaires and physical examination data.

### 2.4 LE8 calculation

This study quantitatively assessed CVH based on AHA's updated standardized algorithm (12). As shown in Supplementary Table S1, both health behavior metrics (diet, physical activity, smoking, sleep) and health factor metrics (BMI, non-HDL cholesterol, blood glucose, and blood pressure) were quantified on a 0–100 scale. For health behavior scores, diet was scored using the HEI-2015 scale based on population percentiles, physical activity was based on weekly exercise duration, smoking was evaluated according to smoking status and household exposure, and sleep was scored based on sleep duration (13). For health factor scores, BMI was based on weight classification, non-HDL cholesterol and blood pressure scores considered relevant medication use, and blood glucose scores incorporated diabetes history and HbA1c levels. The overall CVH score (0–100 points) was calculated by averaging all metric scores, enabling standardized CVH assessment.

### 2.5 Statistical analysis

#### 2.5.1 Statistical analysis models

All statistical analyses incorporated weighting, stratification, and clustering methods to account for NHANES' complex sampling design. Continuous variables are presented as mean ± standard deviation (SD), and categorical variables as frequencies and percentages. Statistical significance was set at *P* < 0.05. Pesticide biomarker concentrations underwent natural logarithmic transformation to improve normality due to their skewed distribution. Each pesticide biomarker concentration was categorized into quartiles (Q1–Q4), with Q1 serving as the reference group. Three progressive multivariate regression models were constructed to analyze associations between pesticide exposure and cardiovascular health: Model 1 (unadjusted); Model 2 (adjusted for age, sex, and race); and Model 3 (further adjusted for education level, income-to-poverty ratio, and cardiovascular disease history including heart failure, coronary heart disease, angina, myocardial infarction, and stroke). To ensure model validity, we conducted residual diagnostics for each regression model, examining assumptions of normality and homoscedasticity; assessed multicollinearity using Variance Inflation Factors (VIF); and evaluated model goodness-of-fit by comparing Bayesian Information Criterion (BIC) values.

#### 2.5.2 Dose-response relationship analysis

To explore potential non-linear relationships between pesticide exposure levels and cardiovascular health indicators, Restricted Cubic Spline (RCS) modeling was employed with five knots, using median exposure levels as reference values. The exposure-response relationship's non-linear characteristics were evaluated by comparing linear models with RCS models, calculating overall effect *P*-values and non-linearity test *P*-values for statistical significance assessment (14, 15). Following Harrell's recommendations, the RCS model established 5 knots at the 5th, 25th, 50th, 75th, and 95th percentiles. Model validation included: comparing linear vs. non-linear models using likelihood ratio tests; calculating AIC and BIC to assess fit quality; and evaluating curve estimation stability through Bootstrap methods (1,000 repetitions). To test robustness, we also examined model performance with varying knot numbers (3, 4, and 5 knots).

#### 2.5.3 Weighted quantile sum (WQS) regression analysis

Weighted Quantile Sum (WQS) regression analysis was used to evaluate the composite effect of pesticide mixture exposure on cardiovascular health and the relative contribution of each component (16). Missing data were handled through multiple imputation, and the dataset was randomly divided into training (40%) and validation (60%) sets. In WQS modeling, five pesticide biomarkers (2,4-D herbicides, atrazine metabolites, organophosphate dimethyl and diethyl metabolites, and glyphosate) were included as continuous variables, with weights for each pesticide in the mixture estimated through 1,000 Bootstrap repetitions. Three progressive models (Models 1–3) were constructed. Model significance was evaluated on the validation dataset, analyzing mixture associations under both positive and negative constraints. WQS regression model validation included: ensuring similar population characteristic distributions between training (40%) and validation (60%) sets; assessing weight estimation stability by comparing coefficients of variation in the Bootstrap sampling distributions; and calculating calibration metrics on the validation set to evaluate predictive performance. We also conducted sensitivity analyses by excluding highly correlated pesticide variables to evaluate the impact of inter-pesticide correlations on WQS weights.

#### 2.5.4 Subgroup analysis

Stratified analyses assessed effect heterogeneity across population subgroups, examining age (20–39, 40–59, ≥60 years), sex (male, female), and race/ethnicity. Survey-weighted logistic regression models calculated associations (OR and 95% CI) within subgroups, with interaction term testing (likelihood ratio test) evaluating effect differences (*P*-interaction) (17, 18). In subgroup analyses, we first ensured adequate sample sizes within each subgroup (at least 10% of the total sample per group); visually assessed effect size differences between subgroups using forest plots; and evaluated the importance of interaction effects by comparing AIC values between models with and without interaction terms.

#### 2.5.5 Mediation analysis

Mediation analysis evaluated the mediating roles of inflammatory markers (NLR, SII, CRP) in pesticide exposure's effects on cardiovascular health. Bootstrap methods (1,000 repetitions) estimated direct effects (ADE), indirect effects (ACME), and their 95% confidence intervals, calculating the proportion of mediation effects (19). All models adjusted for confounding factors including age, sex, race, education level, and income-to-poverty ratio. Validation steps for mediation analysis included: verifying Baron and Kenny mediation conditions to ensure necessary associations between exposure, mediator, and outcome variables; conducting sensitivity analyses to assess potential influences of unmeasured confounders; and testing the robustness of mediation effect estimates by transforming the measurement scale of mediator variables (e.g., logarithmic transformation).

#### 2.5.6 Sensitivity analysis

Sensitivity analyses assessed result robustness through unweighted analysis methods. Using identical model specifications as the main analysis but without NHANES sampling weights, weighted and unweighted results were compared to verify primary findings' reliability. Additionally, three progressive models with consistent covariate adjustment strategies were constructed for primary exposure and outcome variables to evaluate the impact of different adjustment strategies. Beyond comparing weighted and unweighted results, our sensitivity analyses included: incrementally adding potential confounding variables to models to assess core association stability; comparing results across different missing data handling methods (complete case analysis and multiple imputation); and re-evaluating association patterns using alternative pesticide exposure classification methods (tertiles, quintiles). Result stability was assessed through percentage changes in effect sizes and confidence interval overlap.

## 3 Results

### 3.1 Baseline characteristics of study population

The study included 12,432 participants, with 300 cardiovascular deaths and 12,132 non-cardiovascular deaths (Table 1). Regarding demographic characteristics, the study population was predominantly middle-aged, with 38.9% aged 40–59 years. However, in the cardiovascular death group, the proportion of elderly individuals (≥60 years) was significantly higher (79.7% vs. 24.9%, *P* < 0.001). Women comprised 52.1% of the total population but represented a lower proportion in the cardiovascular death group (40.1% vs. 52.4%, *P* < 0.001). Non-Hispanic White people constituted the majority (70.1%) of the racial composition, with an even higher proportion in the cardiovascular death group (82.4%).

**Table 1:** Characteristics of the study participants: NHANES 2007–2018.

**Characteristics**	**Level**	**Overall (*N* = 12,432)**	**Cardiovascular death (*N* = 300)**	**Non-cardiovascular death (*N* = 12,132)**	***P* value**
Age (%)	20–39	4,101 (35.2%)	10 (3.4%)	4,091 (35.8%)	< 0.001
	40–59	4,236 (38.9%)	38 (16.9%)	4,198 (39.3%)	
	≥60	4,095 (25.8%)	252 (79.7%)	3,843 (24.9%)	
Gender (%)	Male	5,951 (47.9%)	183 (59.9%)	5,768 (47.6%)	< 0.001
	Female	6,481 (52.1%)	117 (40.1%)	6,364 (52.4%)	
Race (%)	Mexican American	1,759 (7.6%)	14 (2.7%)	1,745 (7.7%)	< 0.001
	Other Hispanic	1,211 (5.2%)	17 (2.0%)	1,194 (5.3%)	
	Non-Hispanic White	5,698 (70.1%)	201 (82.4%)	5,497 (69.9%)	
	Non-Hispanic Black	2,523 (10.3%)	63 (10.8%)	2,460 (10.3%)	
	Other	1,241 (6.8%)	5 (2.1%)	1,236 (6.8%)	
Education_new (%)	Under high school	2,776 (14.5%)	89 (25.5%)	2,687 (14.3%)	< 0.001
	High school or equivalent	2,788 (22.0%)	89 (28.9%)	2,699 (21.9%)	
	Above high school	6,868 (63.6%)	122 (45.6%)	6,746 (63.9%)	
Pir (%)	< 1	2,570 (13.6%)	55 (12.9%)	2,515 (13.6%)	
	1–3	5,122 (34.8%)	163 (51.7%)	4,959 (34.5%)	
	>3	4,740 (51.6%)	82 (35.4%)	4,658 (51.9%)	
Life_essential_8_score (%)	Low	2,502 (16.1%)	100 (30.7%)	2,402 (15.8%)	< 0.001
	Moderate	8,415 (68.9%)	190 (66.5%)	8,225 (68.9%)	
	High	1,515 (15.1%)	10 (2.8%)	1,505 (15.3%)	
BP_score [mean (SD)]		69.82 (0.50)	45.18 (2.72)	70.25 (0.50)	< 0.001
Diet_score [mean (SD)]		39.87 (0.58)	40.30 (2.42)	39.87 (0.58)	0.858
Blood_glucose_score [mean (SD)]		83.40 (0.29)	66.57 (1.83)	83.69 (0.29)	< 0.001
Smoke_score [mean (SD)]		72.52 (0.71)	69.93 (2.91)	72.57 (0.72)	0.374
Non_HDL_score [mean (SD)]		64.33 (0.38)	61.24 (1.71)	64.39 (0.39)	0.078
BMI_score [mean (SD)]		60.38 (0.46)	57.25 (2.92)	60.44 (0.47)	0.283
Sleep_score (mean (SD))		83.38 (0.33)	77.71 (2.16)	83.48 (0.34)	0.01
PA_score [mean (SD)]		42.19 (0.70)	29.66 (4.22)	42.41 (0.70)	0.003
CMI [mean (SD)]		1.71 (0.04)	1.91 (0.22)	1.71 (0.04)	0.348

Regarding socioeconomic characteristics, 63.6% had above high school education, but this percentage was significantly lower in the cardiovascular death group (45.6% vs. 63.9%, *P* < 0.001). For income levels, 51.6% of the population had an income-to-poverty ratio >3, while this proportion was only 35.4% in the cardiovascular death group.

Concerning cardiovascular health indicators, most participants (68.9%) had moderate LE8 scores, but the cardiovascular death group showed a significantly higher proportion of low scores (30.7% vs. 15.8%, *P* < 0.001). Specifically, the cardiovascular death group performed worse on multiple LE8 components, particularly blood pressure scores (45.18 vs. 70.25, *P* < 0.001), blood glucose scores (66.57 vs. 83.69, *P* < 0.001), sleep scores (77.71 vs. 83.48, *P* = 0.01), and physical activity scores (29.66 vs. 42.41, *P* = 0.003). Regarding CMI, the cardiovascular death group showed slightly higher values than the non-cardiovascular death group (1.91 vs. 1.71), though the difference was not statistically significant (*P* = 0.348).

### 3.2 Correlation analysis of pesticide biomarkers

**Figure 1:**
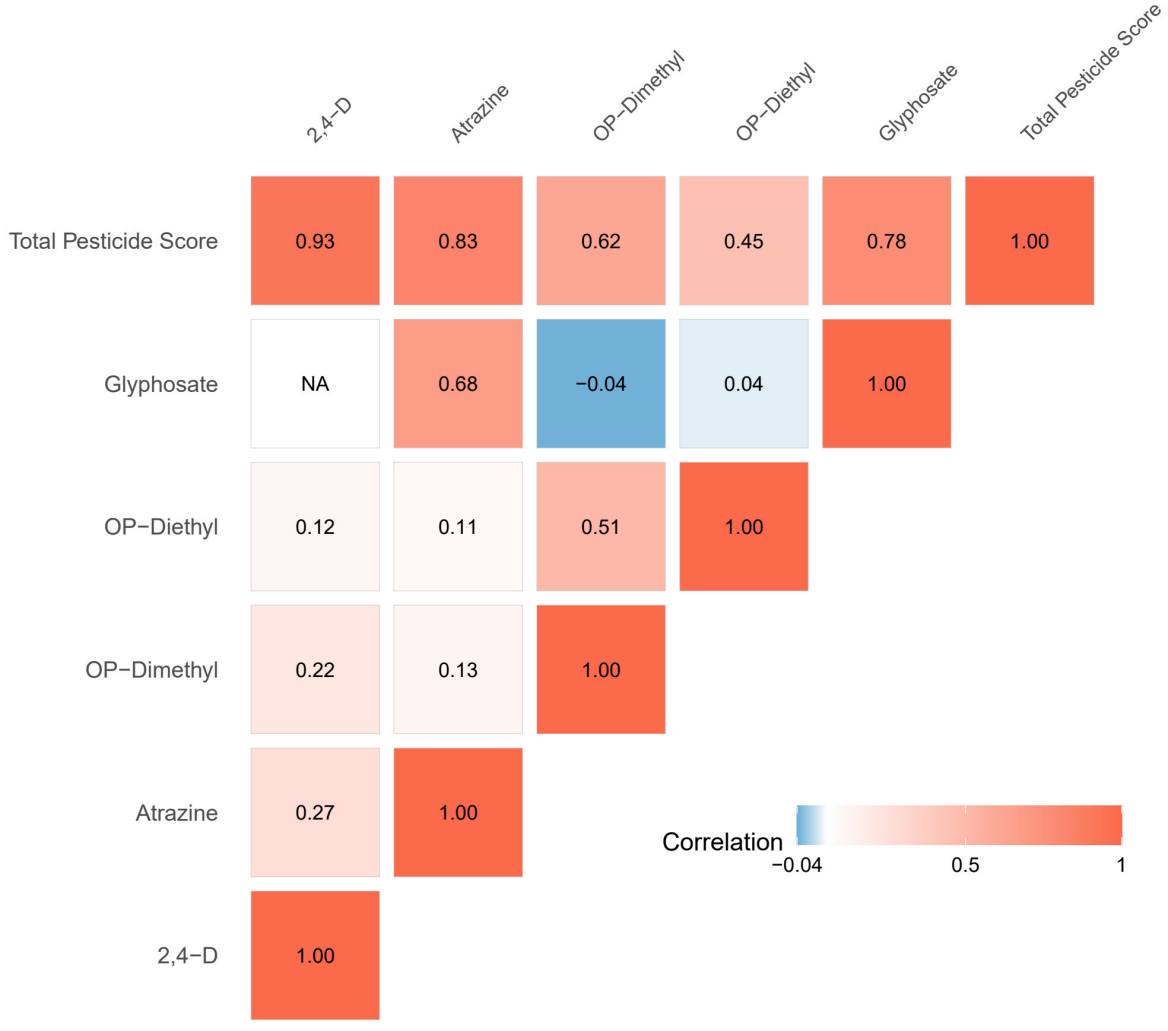
Spearman correlation analysis heatmap.

Spearman correlation analysis revealed generally weak correlations among the five pesticide biomarkers included in the study (Figure 1). 2,4-D class herbicides showed low correlation coefficients with other pesticides, exhibiting weak correlations with atrazine metabolites (*r* = 0.27), organophosphate dimethyl (*r* = 0.22), and diethyl metabolites (*r* = 0.12). A relatively stronger correlation was observed between organophosphate dimethyl and diethyl metabolites (*r* = 0.51), possibly due to their being metabolites of the same class of pesticides. Glyphosate showed moderate correlation only with atrazine metabolites (*r* = 0.68), while showing almost no correlation with organophosphate metabolites (*r* = −0.04 to 0.04).

### 3.3 Weighted regression analysis of pesticide exposure and cardiovascular health indicators

The study evaluated associations between pesticide exposure and cardiovascular health using two approaches: first, analyzing continuous relationships between pesticide biomarkers and cardiovascular health indicators, and second, assessing impacts of different exposure levels through quartile analysis (see Tables 2, 3). Results from Models 1 and 2 for continuous relationships are shown in Supplementary Table S2.

**Table 2:** Associations between pesticide exposure biomarkers and cardiovascular health components in Model 3.

**Variables**	**2,4-D**		**Atrazine**		**OP-Dimethyl**		**OP-Diethyl**		**Glyphosate**		**Total pesticide score**	
	β **(95% Cl)**	***P*** **value**	β **(95% Cl)**	***P*** **value**	β **(95% Cl)**	***P*** **value**	β **(95% Cl)**	***P*** **value**	β **(95% Cl)**	***P*** **value**	β **(95% Cl)**	***P*** **value**
**Continuous (Ln-transformed)**												
BP score	0.627 (0.130, 1.124)	0.016	0.568 (0.040, 1.095)	0.039	0.935 (-0.090, 1.960)	0.080	1.484 (0.057, 2.912)	0.047	−0.037 (−1.239, 1.165)	0.952	0.803 (0.372, 1.234)	< 0.001
Blood glucose score	−0.133 (−0.644, 0.377)	0.611	0.130 (−0.234, 0.494)	0.485	−0.075 (−0.777, 0.626)	0.835	−0.085 (−0.947, 0.776)	0.847	0.321 (−0.352, 0.995)	0.355	−0.141 (−0.490, 0.208)	0.431
Smoke score	−0.135 (−1.014, 0.744)	0.764	−1.451 (−2.100, −0.802)	< 0.001	2.688 (1.367, 4.009)	< 0.001	2.011 (0.079, 3.942)	0.047	−2.865 (−4.641, −1.090)	0.003	−0.265 (−0.920, 0.391)	0.431
Non-HDL score	−0.037 (−0.668, 0.593)	0.908	−0.387 (−1.000, 0.225)	0.220	0.526 (−0.603, 1.655)	0.366	−1.025 (−2.417, 0.367)	0.156	−1.645 (−2.907, −0.384)	0.014	−0.351 (−0.861, 0.159)	0.181
BMI score	−1.441 (−2.158, −0.725)	< 0.001	−1.180 (−1.840, −0.519)	0.001	0.899 (−0.127, 1.925)	0.093	3.503 (1.685, 5.321)	< 0.001	−0.841 (−2.066, 0.384)	0.185	−0.919 (−1.386, −0.452)	< 0.001
Sleep score	−0.617 (−1.107, −0.126)	0.017	−0.004 (−0.428, 0.419)	0.984	−0.395 (−1.303, 0.514)	0.399	−1.412 (−2.619, −0.205)	0.026	0.081 (−1.043, 1.206)	0.888	−0.469 (−0.857, −0.081)	0.020
PA score	−0.026 (−0.949, 0.897)	0.956	2.166 (1.173, 3.158)	< 0.001	−0.710 (−2.126, 0.706)	0.331	−2.325 (−4.765, 0.114)	0.068	3.779 (1.833, 5.724)	< 0.001	0.629 (−0.122, 1.380)	0.105
Diet score	−1.241 (−1.825, −0.658)	< 0.001	−0.865 (−1.462, −0.269)	0.006	4.202 (2.899, 5.506)	< 0.001	5.159 (3.331, 6.987)	< 0.001	−0.278 (−1.400, 0.845)	0.630	−0.160 (−0.663, 0.343)	0.534
LE8 score	−0.375 (−0.623, −0.128)	0.004	−0.128 (−0.414, 0.158)	0.383	1.009 (0.539, 1.478)	< 0.001	0.914 (0.195, 1.633)	0.016	−0.186 (−0.714, 0.343)	0.494	−0.109 (−0.320, 0.102)	0.315
CMI	0.017 (−0.040, 0.073)	0.565	−0.022 (−0.072, 0.027)	0.380	−0.055 (−0.164, 0.055)	0.334	0.126 (−0.028, 0.279)	0.115	−0.009 (−0.109, 0.091)	0.859	−0.010 (−0.058, 0.039)	0.700

**Table 3:** Dose-response relationship between pesticide exposure biomarkers and Life's Essential 8 score.

**Pesticides**	**Quartiles**	**Crude (β, 95% Cl)**	***P* value**	**Model 2 (β, 95% Cl)**	***P* value**	**Model 3 (β, 95% Cl)**	***P* value**
2,4-D	Q1	Ref		Ref		Ref	
	Q2	−1.049 (−2.485, 0.386)	0.156	−0.803 (−2.168, 0.561)	0.253	−0.593 (−1.859, 0.672)	0.362
	Q3	−2.628 (−4.060,−1.195)	0.001	−1.892 (−3.317,−0.467)	0.011	−1.445 (−2.740,−0.150)	0.033
	Q4	−3.578 (−4.963, −2.193)	< 0.001	−2.203 (−3.616, −0.790)	0.003	−1.204 (−2.405, −0.003)	0.054
Atrazine	Q1	Ref		Ref		Ref	
	Q2	−0.791 (−1.932, 0.350)	0.178	−0.794 (−1.897, 0.310)	0.163	−0.290 (−1.360, 0.781)	0.598
	Q3	−1.739 (−3.042, −0.437)	0.011	−1.906 (−3.187, −0.625)	0.005	−1.252 (−2.449, −0.055)	0.045
	Q4	−1.757 (−3.359, −0.155)	0.035	−1.800 (−3.244, −0.356)	0.017	−0.959 (−2.356, 0.438)	0.184
OP-Dimethyl	Q1	Ref		Ref		Ref	
	Q2	0.872 (−0.639, 2.384)	0.262	1.220 (−0.165, 2.606)	0.090	0.891 (−0.471, 2.254)	0.207
	Q3	2.147 (0.700, 3.594)	0.005	2.604 (1.212, 3.996)	0.001	2.033 (0.585, 3.482)	0.009
	Q4	3.632 (2.052, 5.212)	< 0.001	4.581 (3.110, 6.052)	< 0.001	3.400 (1.940, 4.859)	< 0.001
OP-Diethyl	Q1	Ref		Ref		Ref	
	Q2	0.745 (−0.944, 2.434)	0.391	1.272 (−0.359, 2.902)	0.132	0.777 (−0.748, 2.302)	0.323
	Q3	1.319 (−0.553, 3.192)	0.172	1.500 (−0.294, 3.294)	0.107	1.108 (−0.605, 2.821)	0.212
	Q4	2.119 (0.203, 4.034)	0.034	2.444 (0.565, 4.323)	0.014	2.130 (0.438, 3.823)	0.018
Glyphosate	Q1	Ref		Ref		Ref	
	Q2	1.042 (−1.074, 3.158)	0.338	1.305 (−0.806, 3.416)	0.231	0.981 (−0.786, 2.748)	0.282
	Q3	1.065 (−1.104, 3.234)	0.340	1.598 (−0.637, 3.833)	0.167	1.240 (−0.601, 3.081)	0.193
	Q4	0.140 (−1.941, 2.220)	0.896	0.447 (−1.679, 2.573)	0.682	0.436 (−1.389, 2.261)	0.642
Total pesticide score	Q1	Ref		Ref		Ref	
	Q2	−0.128 (−1.152, 0.896)	0.807	−0.069 (−1.027, 0.889)	0.888	0.052 (−0.872, 0.976)	0.912
	Q3	0.621 (−0.336, 1.579)	0.207	0.710 (−0.234, 1.654)	0.144	0.767 (−0.099, 1.633)	0.087
	Q4	−1.509 (−2.553, −0.464)	0.006	−0.899 (−1.935, 0.136)	0.092	−0.377 (−1.317, 0.563)	0.434

After adjusting for demographic characteristics, socioeconomic status, and cardiovascular disease history, different pesticide biomarkers showed varying association patterns with cardiovascular health indicators (Table 2):

2,4-D herbicides showed significant negative correlations with multiple LE8 components, including BMI scores (β = −1.441, 95% CI: −2.158, −0.725), sleep scores (β = −0.617, 95% CI: −1.107, −0.126), and diet scores (β = −1.241, 95% CI: −1.825, −0.658), but positive correlation with blood pressure scores (β = 0.627, 95% CI: 0.130, 1.124).

Organophosphate metabolites (dimethyl and diethyl) exposure showed significant positive correlations with smoking scores, diet scores, and total LE8 scores. Dimethyl metabolites showed strongest associations with smoking scores (β = 2.688, 95% CI: 1.367, 4.009) and diet scores (β = 4.202, 95% CI: 2.899, 5.506), while diethyl metabolites showed strongest positive correlation with BMI scores (β = 3.503, 95% CI: 1.685, 5.321).

Glyphosate showed significant positive correlation with physical activity scores (β = 3.779, 95% CI: 1.833, 5.724), but negative correlations with smoking scores (β = −2.865, 95% CI: −4.641, −1.090) and non-HDL scores (β = −1.645, 95% CI: −2.907, −0.384).

Total pesticide exposure scores showed significant positive correlation with blood pressure scores (β = 0.803, 95% CI: 0.372, 1.234) and negative correlations with BMI scores (β = −0.919, 95% CI: −1.386, −0.452) and sleep scores (β = −0.469, 95% CI: −0.857, −0.081). Notably, no significant associations were found between any pesticide exposure indicators and CMI.

Quartile analysis (Table 3) revealed significant differences in association patterns between exposure levels of different pesticides and LE8 scores. 2,4-D herbicides and atrazine metabolites showed negative correlations, with Q3 groups showing significant negative associations compared to Q1 groups in fully adjusted models (2,4-D: β = −1.445, 95% CI: −2.740, −0.150; atrazine: β = −1.252, 95% CI: −2.449, −0.055).

In contrast, organophosphate metabolites showed positive associations. Dimethyl metabolites showed significant positive correlations in Q3 (β = 2.033, 95% CI: 0.585, 3.482) and Q4 groups (β = 3.400, 95% CI: 1.940, 4.859), with stronger associations at higher exposure levels. Diethyl metabolites showed significant positive association in Q4 group (β = 2.130, 95% CI: 0.438, 3.823).

Glyphosate metabolites and total pesticide exposure scores showed no significant statistical relationships with LE8 scores. These associations persisted after adjustment for potential confounders, though with reduced strength. While glyphosate metabolites showed no significant association with LE8 scores, they showed significant negative correlation with CMI at Q2 exposure level (β = −0.437, 95% CI: −0.836, −0.038). Only glyphosate showed correlations with CMI (Supplementary Table S3).

**Figure 2:**
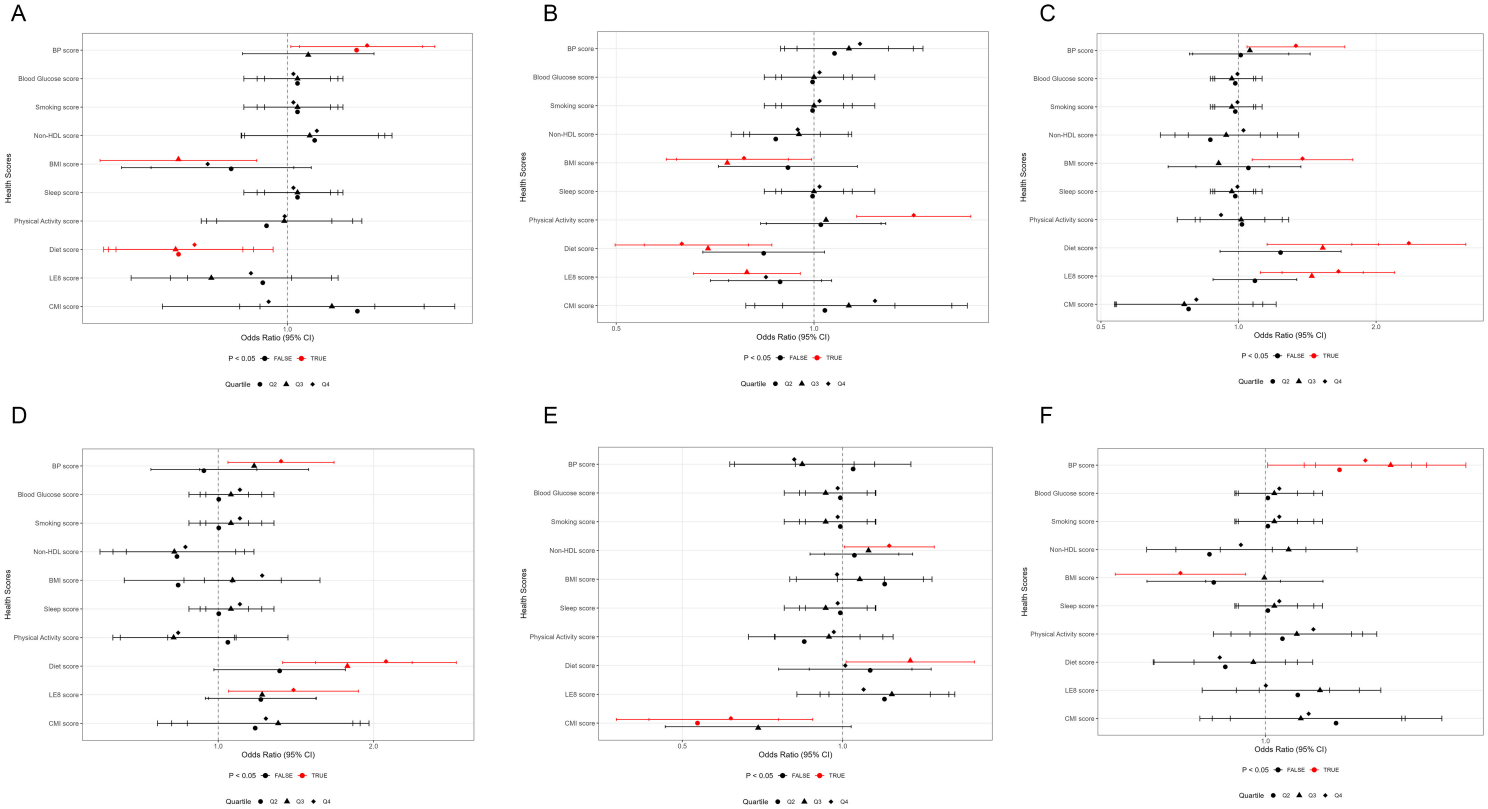
Associations between pesticide exposure biomarkers and cardiovascular health indicators. **(A)** Associations between 2,4-D and health scores. **(B)** Associations between Atrazine and health scores. **(C)** Associations between OP-Dimethyl and health scores. **(D)** Associations between OP-Diethyl and health scores. **(E)** Associations between Glyphosate and health scores. **(F)** Associations between Total Pesticide Score and health scores.

Weighted logistic regression further validated the associations between pesticide exposure and cardiovascular health indicators (Figure 2). Results showed that certain associations-maintained consistency with continuous variable analyses:

Organophosphate dimethyl metabolites showed significant positive correlation with high-level LE8, with Q3 group (OR = 1.45, 95% CI: 1.12–1.87) and Q4 group (OR = 1.65, 95% CI: 1.25–2.20) both showing higher probability of good cardiovascular health. Similarly, the Q4 group of organophosphate diethyl metabolites also demonstrated significant positive association (OR = 1.40, 95% CI: 1.05–1.87).

Regarding dietary behaviors, the positive associations with organophosphate pesticides were also confirmed, with dimethyl Q4 group (OR = 2.36, 95% CI: 1.77–3.14) and diethyl Q4 group (OR = 2.11, 95% CI: 1.54–2.89) both significantly increasing the likelihood of good dietary behaviors. In contrast, 2,4-D and atrazine exposure were associated with poorer dietary behaviors, consistent with continuous variable analysis results.

For blood pressure control, the positive association with total pesticide exposure score was confirmed, with Q2 to Q4 groups all showing significantly increased hypertension risk (ORs ranging from 1.19 to 1.34). Interestingly, glyphosate was the only pesticide showing significant association with CMI, with both Q2 group (OR = 0.53, 95% CI: 0.38–0.76) and Q4 group (OR = 0.62, 95% CI: 0.43–0.88) reducing the risk of high CMI.

### 3.4 Dose-response relationship between individual pesticides exposure and health scores

**Figure 3:**
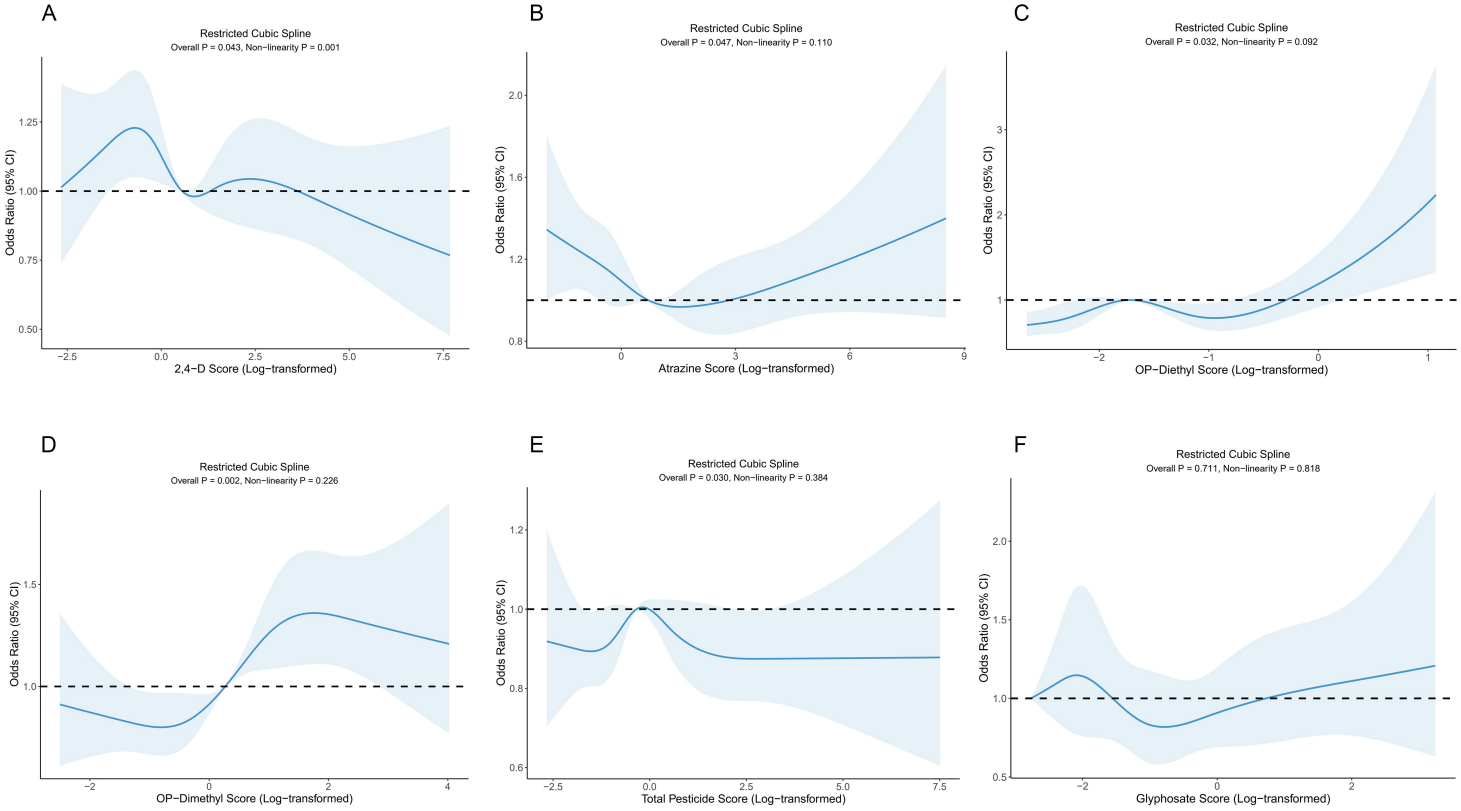
Restricted cubic spline analysis of associations between pesticide exposures and LE8 scores. **(A)** Association between 2,4-D and LE8 scores. **(B)** Association between Atrazine and LE8 scores. **(C)** Association between OP-Diethyl and LE8 scores. **(D)** Association between OP-Dimethyl and LE8 scores. **(E)** Association between Total Pesticide Score and LE8 scores. **(F)** Association between Glyphosate and LE8 scores.

Restricted cubic spline (RCS) analysis was used to evaluate relationships between various pesticide exposures and LE8 scores (Figure 3).

2,4-D herbicides showed significant non-linear association with LE8 scores (overall *P* = 0.043, non-linearity *P* = 0.001). The dose-response curve showed an inflection point at a log-transformed exposure level of −0.341, displaying an “inverted U-shape” relationship, suggesting the existence of an optimal exposure level.

Organophosphate pesticide metabolites both showed overall significance (dimethyl: *P* = 0.002; diethyl: *P* = 0.032), but did not exhibit significant non-linear characteristics (dimethyl: non-linearity *P* = 0.226; diethyl: non-linearity *P* = 0.092). The dimethyl metabolites showed an inflection point at log value 0.269, followed by a stable positive correlation trend; diethyl metabolites showed an inflection point at log value −1.729, followed by a gradually increasing trend.

Atrazine also showed statistically significant association with LE8 scores (overall *P* = 0.047, non-linearity *P* = 0.110), with an inflection point at log value 0.708, followed by a slow upward trend. Total pesticide exposure score also showed significant overall association (*P* = 0.030) but did not exhibit obvious non-linear characteristics (non-linearity *P* = 0.384).

### 3.5 Mixed exposure effect analysis

**Figure 4:**
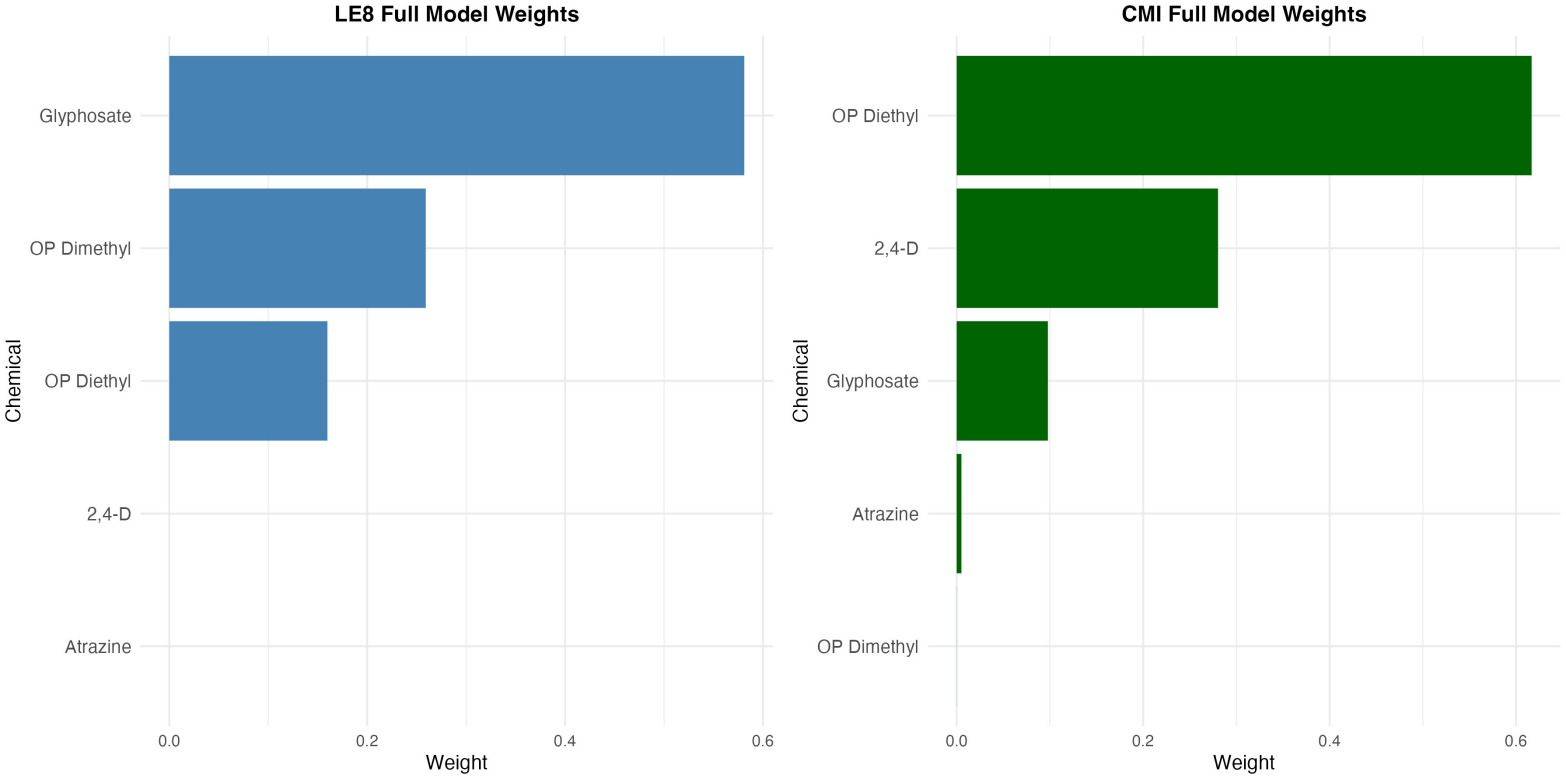
Weighted contributions of individual pesticides in the WQS regression models.

Weighted quantile sum (WQS) regression analysis was used to evaluate associations between mixed pesticide exposure and cardiovascular health (Figure 4). Results showed significant associations between mixed pesticide exposure and both LE8 scores and CMI, which remained stable across different adjustment models.

In LE8 score analysis, the fully adjusted model showed the strongest association (β = 0.828, 95% CI: 0.579–1.077, *P* < 0.001), with glyphosate contributing the largest weight (~58%), followed by organophosphate dimethyl metabolites (~26%) and diethyl metabolites (~16%). In CMI analysis, although the overall association was relatively weak, it remained statistically significant (β = 0.063, 95% CI: 0.031–0.096, *P* < 0.001), with a different weight distribution pattern: organophosphate diethyl metabolites dominated (~62%), followed by 2,4-D (~28%), while glyphosate (~10%), atrazine, and organophosphate dimethyl metabolites contributed less.

### 3.6 Subgroup analysis results

Subgroup analyses further revealed population heterogeneity in associations between pesticide exposure and cardiovascular health (Supplementary Table S4). Interaction analysis showed that, except for the interaction between glyphosate and gender (*P*-interaction = 0.037), other interactions between pesticide exposure and age, gender, and race did not reach statistical significance.

Age-stratified analysis showed that organophosphate dimethyl metabolites demonstrated significant positive associations across all age groups (20–39 years: OR = 1.195, 95% CI: 1.051–1.358; 40–59 years: OR = 1.269, 95% CI: 1.061–1.516; ≥60 years: OR = 1.261, 95% CI: 1.083–1.469), while 2,4-D showed significant negative association only in young populations (20–39 years) (OR = 0.807, 95% CI: 0.703–0.927).

Gender-stratified analysis found that organophosphate dimethyl metabolites showed significant positive associations in both males (OR = 1.219, 95% CI: 1.078–1.379) and females (OR = 1.275, 95% CI: 1.111–1.462), while 2,4-D showed significant negative association only in females (OR = 0.810, 95% CI: 0.713–0.922).

Race-stratified analysis indicated that organophosphate diethyl metabolites showed the strongest positive association in Mexican Americans (OR = 1.304, 95% CI: 1.113–1.527), while organophosphate dimethyl metabolites showed the most significant association in non-Hispanic White people (OR = 1.317, 95% CI: 1.161–1.495). Meanwhile, 2,4-D in non-Hispanic White people (OR = 0.892, 95% CI: 0.804–0.988), and glyphosate and atrazine in Mexican Americans (OR = 0.743, 95% CI: 0.574–0.961; OR = 0.785, 95% CI: 0.663-0.929) all showed significant negative associations.

### 3.7 Mediation analysis results

Mediation analysis for 2,4-D exposure showed significant mediation through CRP (ACME = −0.003, 95% CI: −0.007 to −0.002, *P* < 0.001), accounting for 20.3% of the total effect. SII also showed a weak but significant mediating effect (ACME = −0.005, 95% CI: −0.002 to −0.0001, *P* = 0.028).

Among organophosphate pesticides, diethyl metabolites showed significant mediation through SII (ACME = −0.007, 95% CI: −0.008 to −0.002, *P* = 0.002), accounting for 36.8% of the total effect, while also showing significant direct effects (ADE = 0.027, 95% CI: 0.008–0.047, *P* = 0.008). Dimethyl metabolites primarily showed direct effects (ADE = 0.032, 95% CI: 0.017–0.049, *P* < 0.001), with no significant mediation through inflammation markers.

Glyphosate exposure primarily showed mediation through CRP (ACME = −0.007, 95% CI: −0.010 to −0.001, *P* = 0.012). Total pesticide exposure score also showed significant mediation through CRP (ACME = −0.004, 95% CI: −0.004 to −0.001, *P* < 0.001), with all inflammation markers showing weak but statistically significant mediation effects (*P* < 0.05 for both NLR and SII).

No significant mediating effects were observed between atrazine exposure and inflammation markers, suggesting it might affect cardiovascular health through other mechanisms. These results indicate that inflammatory response may be an important biological pathway through which some pesticide exposures affect cardiovascular health, though different types of pesticides may act through different mechanisms.

### 3.8 Sensitivity analysis

Supplementary Table S5 showed the results of sensitivity analysis after multiple imputation. In Model 3, the positive associations between organophosphate pesticides (diethyl metabolites β = 1.985, 95% CI: 1.601–2.368 and dimethyl metabolites β = 1.625, 95% CI: 1.356–1.895) and LE8 scores, and the negative associations of 2,4-D (β = −0.635, 95% CI: −0.812 to −0.458) and atrazine (β = −0.408, 95% CI: −0.574 to −0.242) remained consistent. Glyphosate and total pesticide exposure score still showed no significant associations after imputation.

## 4 Discussion

This study marks the first systematic evaluation of the associations between pesticide exposure and both non-cardiovascular mortality and cardiovascular health in the general population. Through analysis of data from 12,432 adult participants in the NHANES 2007–2018 database, we identified significant differential effects of various pesticide types on cardiovascular health. 2,4-D herbicides showed significant negative correlations with multiple LE8 components, including BMI score, sleep score, and dietary score; organophosphate pesticide metabolites (dimethyl and diethyl) generally demonstrated positive associations with cardiovascular health indicators, particularly evident in smoking score, dietary score, and total LE8 score. Dose-response analysis revealed complex non-linear relationships, with 2,4-D herbicides exhibiting an “inverted U-shaped” relationship with LE8 scores, showing an inflection point at the exposure level logarithm of −0.341; meanwhile, organophosphate pesticide metabolites displayed relatively stable positive correlation trends. In mixed exposure effect analysis, WQS regression results indicated that glyphosate contributed the highest weight (~58%) to LE8 scores, while organophosphate diethyl metabolites dominated in CMI analysis (~62%). Furthermore, mediation analysis revealed that inflammatory markers (particularly CRP and SII) play crucial roles in the process by which pesticide exposure affects cardiovascular health, providing new perspectives for understanding the underlying mechanisms.

Our findings reveal several clinically significant associations with important public health implications. Among the examined pesticide biomarkers, 2,4-D herbicides demonstrated the most consistent negative associations with cardiovascular health, particularly affecting BMI and diet scores. The inverted U-shaped relationship between 2,4-D exposure and LE8 scores suggests there is no 'safe' threshold for exposure, as even low-level exposure shows significant negative associations with cardiovascular health. In contrast, organophosphate metabolites showed unexpected positive associations with LE8 scores, which may reflect their correlation with certain healthy dietary patterns or potential survival bias in our study population. The substantial mediating role of inflammatory markers provides a plausible biological pathway connecting pesticide exposure to cardiovascular outcomes and identifies potential intervention targets.

In this study, different types of pesticides demonstrated distinct mechanistic pathways: 2,4-D herbicides and organophosphate pesticides potentially affect cardiovascular health through mitochondrial dysfunction and oxidative stress induction, leading to increased cardiomyocyte apoptosis and endothelial dysfunction (20–23). Glyphosate exhibited a unique mode of action, not only influencing energy metabolism but also interfering with lipid metabolism pathways (24–26). Intriguingly, our results indicated a positive correlation between organophosphate pesticides and LE8 index, which we attribute to two main factors. First, studies have shown that specific populations (such as pregnant women in agricultural regions) exhibit significantly elevated DAP metabolite levels, reaching 2.5 times that of NHANES female participants, closely associated with increased fruit and vegetable consumption. This finding suggests that elevated organophosphate metabolite levels might reflect healthier lifestyle choices, which explains our observed positive correlations including smoking and dietary factors (27–30). Additionally, certain environmental chemicals (such as organophosphate insecticides) tend to accumulate in biological adipose tissues, particularly adipocytes. For populations with higher BMI and advanced age, prolonged exposure time and increased storage result in higher accumulated chemical levels, leading to significantly increased health risks (31–33). In our study, these populations often exhibited cardiovascular disease symptoms earlier or experienced mortality due to cumulative damage, suggesting our observations likely reflect survival bias.

These unique patterns were identified through differences in study population characteristics, exposure assessment methods, and outcome indicator selection. While studies focusing on occupationally exposed populations typically involve high exposure levels and extended durations, our study incorporated the general population, utilizing multiple pesticide biomarkers for comprehensive assessment, potentially better reflecting the health effects of long-term low-dose exposure in the general population. Furthermore, this study integrated both single and mixed exposure effects, employing more sophisticated statistical models to evaluate dose-response relationships. Additionally, most studies focusing on LE8 scores and CMI indicators typically concentrate on single clinical endpoints, an approach often leading to bias when focusing solely on patient populations, which aligns with our findings in Table 1 of the first section. Our study uniquely explored the effects of various pesticides on LE8 scores and CMI indicators in the general population. These methodological innovations enabled a more comprehensive investigation of pesticide exposure's impact on cardiovascular health.

Mediation analysis further revealed the crucial role of inflammatory responses in pesticide exposure's impact on cardiovascular health. CRP demonstrated significant mediation effects (20.3% of total effect), suggesting systemic inflammation's importance in pesticide-induced cardiovascular damage. SII and NLR mediation effects reflected immune system involvement, particularly in organophosphate diethyl metabolites' effects, with SII mediating 36.8% of total effects. These findings suggest pesticide exposure may activate multiple inflammatory pathways, including NF-κB signaling and MAPK cascades, ultimately leading to cardiovascular function alterations (34, 35). Different pesticide types act through distinct signaling pathways and mediators, forming complex network effects.

Subgroup analyses revealed significant population heterogeneity in pesticide exposure-cardiovascular health associations. Age-stratified analysis showed organophosphate dimethyl metabolites maintained significant positive associations across all age groups, while 2,4-D showed significant negative associations only in young adults (20–39 years). These age-related differences may reflect the impact of metabolic capacity and detoxification mechanism changes with age, with younger populations potentially more sensitive to certain pesticides. Gender difference analysis found significant interactions only between glyphosate and gender, with other pesticide exposures showing non-significant gender differences. Notably, glyphosate was the only pesticide showing significant associations with CMI, with Q2 (OR = 0.53, 95% CI: 0.38–0.76) and Q4 (OR = 0.62, 95% CI: 0.43–0.88) groups showing reduced high CMI risk. This unique association pattern may relate to glyphosate's specific mechanisms affecting lipid and glucose metabolism, possibly subject to gender-specific regulation, explaining the significant interactions observed in gender-stratified analysis (36, 37). Race-stratified results showed varying organophosphate pesticide effects across races, most pronounced in Mexican Americans and non-Hispanic White people, possibly due to differences in genetic background, lifestyle, and environmental exposure patterns (38). These findings emphasize the need for population-specific considerations in prevention strategy development and differentiated intervention measures for various populations.

This study possesses several notable strengths: it is based on NHANES national survey data with a large sample size, ensuring good population representativeness; it employs multiple pesticide biomarkers for comprehensive assessment, providing a more thorough exposure evaluation; and it utilizes sophisticated statistical methods, including WQS regression and mediation analysis, to thoroughly investigate exposure-response relationships and potential mechanisms. However, the study also has several limitations: the cross-sectional design precludes establishment of causal relationships; single biomarker measurements may not reflect long-term exposure levels; furthermore, several methodological limitations should be acknowledged: the WQS regression, while useful for mixture analysis, assumes similar directional effects across all mixture components and may not fully capture complex interactions between pesticides; RCS analysis is sensitive to knot placement and may not optimally characterize exposure-response relationships with limited data points at extreme exposure levels; mediation analysis relies on untestable assumptions about unmeasured confounding and temporal ordering that cannot be verified in cross-sectional data; and our statistical approaches may be sensitive to sample size variations across subgroups and the handling of missing data despite our validation efforts; additionally, certain potential confounding factors (such as occupational exposure history and dietary habits) may not have been adequately adjusted for. Future research should conduct prospective cohort studies to establish causality, further explore molecular mechanisms, particularly the role of inflammatory pathways, and evaluate the effectiveness of various intervention measures. Additionally, assessment of policy implementation effects is needed to optimize pesticide exposure control strategies.

From a public health perspective, our WQS regression analysis revealed that glyphosate contributed most significantly to overall pesticide mixture effects on LE8 scores, while organophosphate diethyl metabolites dominated CMI effects, highlighting differential impacts across pesticide types. These findings support the need for more stringent regulation of certain pesticides (particularly 2,4-D), pesticide-specific risk assessments rather than treating all agricultural chemicals as homogenous, and targeted public health messaging for vulnerable subpopulations identified in our analysis. Implementation of comprehensive biomonitoring programs could help identify at-risk individuals and track the effectiveness of regulatory interventions aimed at reducing harmful pesticide exposure in the general population.

## 5 Conclusion

In summary, our study provides novel evidence of differential associations between pesticide exposure and cardiovascular health in a nationally representative sample of U.S. adults from NHANES 2007–2018. Specifically, 2,4-D herbicides showed significant negative correlations with multiple LE8 components, while organophosphate metabolites demonstrated protective associations with cardiovascular health indicators. Atrazine exposure showed moderate negative associations with cardiovascular health, particularly impacting BMI and dietary scores, as well as showing positive associations with blood pressure components, though these associations were less consistent than those observed for 2,4-D. The exposure-response analysis revealed a non-linear relationship for 2,4-D (inverted U-shape) and stable positive trends for organophosphate metabolites. In the mixed exposure analysis, glyphosate contributed the highest weight (58%) to LE8 scores, while organophosphate diethyl metabolites dominated the CMI relationship (62%). Notably, inflammatory markers (CRP and SII) were identified as significant mediators in these associations, particularly for 2,4-D and organophosphate exposures. These findings contribute to our understanding of pesticide exposure's impact on cardiovascular health and have important implications for public health interventions. Further research, particularly prospective cohort studies, is warranted to establish causal relationships and explore the underlying molecular mechanisms. Additionally, strengthening environmental monitoring and developing targeted prevention strategies for different population subgroups would be advisable based on our findings of demographic heterogeneity in pesticide exposure effects.

## Data Availability

The original contributions presented in the study are included in the article/Supplementary material, further inquiries can be directed to the corresponding author.
