# Supplementary figures and images for: Pesticide exposure and cardiovascular health in non-CVD mortality population: novel evidence from NHANES 2007–2018 using Life's Essential 8
Source: Front Nutr. 2025 Jun 26;12:1578796. doi: 10.3389/fnut.2025.1578796 (PMC12243112; doi:10.3389/fnut.2025.1578796)

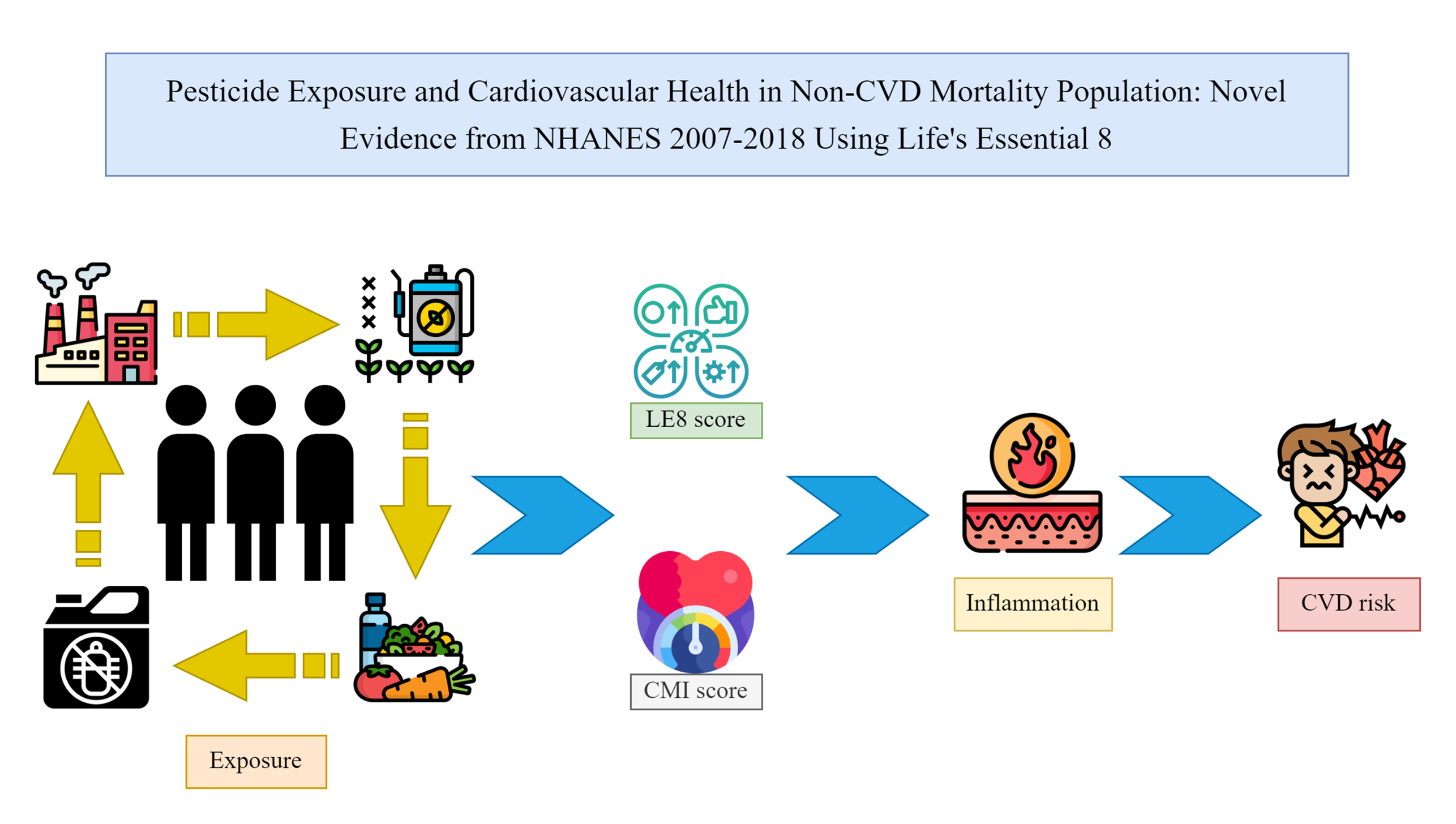

Supplement: Supplementary file 1 [file Image_1.png]
